# Structure and function of TatD exonuclease in DNA repair

**DOI:** 10.1093/nar/gku732

**Published:** 2014-08-11

**Authors:** Yi-Chen Chen, Chia-Lung Li, Yu-Yuan Hsiao, Yulander Duh, Hanna S. Yuan

**Affiliations:** 1 Institute of Molecular Biology, Academia Sinica, Taipei 11529, Taiwan, ROC; 2 Department of Biological Science and Technology, National Chiao Tung University, Hsinchu 30068, Taiwan, ROC; 3 Graduate Institute of Biochemistry and Molecular Biology, College of Medicine, National Taiwan University, Taipei 10048, Taiwan, ROC

## Abstract

TatD is an evolutionarily conserved protein with thousands of homologues in all kingdoms of life. It has been suggested that TatD participates in DNA fragmentation during apoptosis in eukaryotic cells. However, the cellular functions and biochemical properties of TatD in bacterial and non-apoptotic eukaryotic cells remain elusive. Here we show that *Escherichia coli* TatD is a Mg^2+^-dependent 3′–5′ exonuclease that prefers to digest single-stranded DNA and RNA. TatD-knockout cells are less resistant to the DNA damaging agent hydrogen peroxide, and TatD can remove damaged deaminated nucleotides from a DNA chain, suggesting that it may play a role in the H_2_O_2_-induced DNA repair. The crystal structure of the apo-form TatD and TatD bound to a single-stranded three-nucleotide DNA was determined by X-ray diffraction methods at a resolution of 2.0 and 2.9 Å, respectively. TatD has a TIM-barrel fold and the single-stranded DNA is bound at the loop region on the top of the barrel. Mutational studies further identify important conserved metal ion-binding and catalytic residues in the TatD active site for DNA hydrolysis. We thus conclude that TatD is a new class of TIM-barrel 3′–5′ exonuclease that not only degrades chromosomal DNA during apoptosis but also processes single-stranded DNA during DNA repair.

## INTRODUCTION

**Figure 1.**
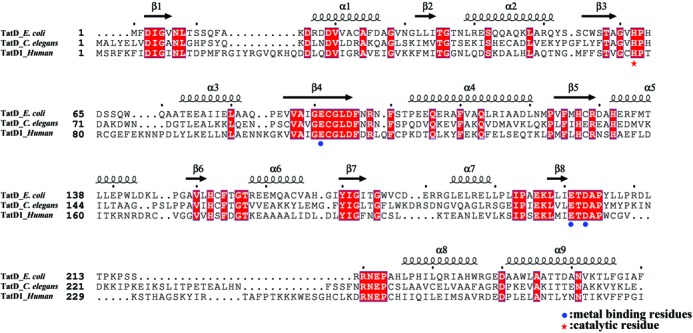
Sequence alignment of TatD. The *E. coli* TatD shares a high sequence identity with *C. elegans* TatD (37%) and human TatD1 (32%). The conserved residues are shaded in red. The secondary structures derived from the crystal structure of TatD (this study, PDB entry: 4P5U) are displayed above the sequences. The conserved metal-ion binding and catalytic residues are marked by blue dots and a red star, respectively.

TatD is a conserved protein widely expressed in different species, including bacteria, fungi, plants and animals. More than 8000 genes encoding TatD-related proteins have been sequenced, mostly bearing a single conserved TatD domain of ∼250 amino acids (see TatD–DNase subfamily in Pfam database, http://pfam.sanger.ac.uk//family/PF01026). TatD from different species share a high sequence identity, such as *Escherichia coli* TatD shares a sequence identity of 26, 37 and 32% to *Saccharomyces cerevisiae*, *Caenorhabditis elegans* and human TatD, respectively, suggesting that TatD may have a conserved structure and function (Figure 1). Nevertheless, little is known regarding either the cellular functions or biochemical properties of TatD in prokaryotic or eukaryotic cells.

TatD was encoded by a *Tat* operon that encodes Tat proteins, including TatA, TatB, TatC and TatD, for protein transport via the Tat (Twin-Arginine Translocation) pathway in *E. coli* (1). TatA, TatB and TatC are membrane-bound proteins and they form a receptor essential for binding and transporting folded proteins bearing twin arginine signal peptides from the cytoplasm to periplasm (2). However, TatD is a cytoplasmic protein with Mg^2+^-dependent DNase activity, and the expression of TatD and two TatD homologues, YcfH and YjjV, are not essential for protein export in the Tat pathway in *E. coli* (3–5). The link between the DNase activity of TatD to protein export seems remote, implying that TatD likely carries an unknown and unrelated cellular function other than protein export in bacteria.

Subsequently, a search of nucleases involved in DNA fragmentation in *C. elegans* identified TatD homologue CRN-2 as one of the apoptotic nucleases (6). Knockdown of CRN-2 delayed DNA degradation of apoptotic cells and increased TUNEL-positive (terminal deoxynucleotidyltransferase-mediated dUTP-nick end labeling) nuclei during development, suggesting that CRN-2 is involved in DNA fragmentation in *C. elegans*. CRN-2 degrades chromosomal DNA and contributes to cell killing in an independent pathway different from that of CPS-6/CRN-1/CRN-4/CRN-5 (7–9). Follow-up studies further showed that TatD bears 3′–5′ exonuclease activity and participates in DNA fragmentation during apoptosis in *S. cerevisiae* (10) and *Trypanosoma brucei* (11). Knockout of TatD increases the TUNEL-positive cells and cell survival whereas overexpression of TatD facilitates cell death in both species, confirming the role of TatD for DNA degradation during apoptosis.

A number of crystal structures of TatD from various species have been deposited in the protein data bank without related published articles, including human TatD1 and TatD3 (PDB entry code: 2XIO and 2YIH), *S. cerevisiae* TatD (PDB entry code: 3E2V) and *E. coli* TatD, YcfH and YjjV (PDB entry code: 1XWY, 1YIX and 1ZZM). These TatD structures all share a similar TIM-barrel fold with eight β/α motifs folded into a barrel-shaped structure. The TIM-barrel fold is one of the most common folds of metabolizing enzymes and can be found in oxireductases, transferases, hydrolases, lyases and isomerases (12). Among the TIM-barrel enzymes, only one nuclease that targets DNA, Endo IV in the AP (apurinic/apyrimidinic) endonuclease family, has been reported (13). Endo IV cleaves DNA at the 5′-side of abasic sites in the base excision DNA repair pathway in a Zn^2+^-dependent manner. The crystal structure of Endo IV bound to DNA reveals that DNA is bound at the C-terminal edge of the β barrel with three zinc ions bound in the endonuclease active site.

Since TatD is an evolutionarily conserved protein, it should have an important cellular role. However, our understanding of this protein is largely hampered due to lack of knowledge of its biological functions and structure-to-function relationship. Here we provide lines of evidence showing that TatD is a 3′–5′ exonuclease that processes single-stranded DNA in DNA repair. The crystal structures of TatD and TatD–DNA complex further reveal how TatD binds and processes DNA from the 3′ end. This study thus reveals, for the first time, the structure and function of TatD in DNA repair at the molecular level.

## MATERIALS AND METHODS

### TatD expression and purification

The gene encoding *E. coli* TatD was amplified by Polymerase Chain Reaction (PCR) using *E. coli* genomic DNA from K12 strains. The PCR products were digested with NdeI and XhoI and inserted in the expression vector pET22b (Novagen) to generate the C-terminal His-tagged recombinant protein. The pET22b-TatD plasmid was transformed into *E. coli* BL21-CodonPlus-RIPL (Stratagene) competent cells cultured in Luria-Bertani (LB) medium supplemented with 100 μg of ampicillin/ml at 37°C. Cells were grown to an OD_600_ of 0.5–0.6 and induced by 1 mM isopropyl-beta-D-thiogalactopyranoside (IPTG) at 18°C for 20 h. The harvested cell pellet was resuspended in the Ni-buffer A (NA) buffer (20 mM 3-(N-morpholino)propanesulfonic acid (MOPS), 300 mM NaCl, pH 7.7) and disrupted by microfluidizer. The crude cell extract was applied to a Ni-NTA column (GE Healthcare) that was washed by NA buffer, and the bound proteins were eluted by an imidazole gradient using a NB buffer (20 mM MOPS, 300 mM NaCl, 500 mM imidazole, pH 7.7) mixing with NA buffer (gradient length: 200 ml). Eluted proteins were dialyzed against QA buffer (20 mM MOPS, 1 mM β-mercaptoethanol, pH 7.2) before being passed through HiTrap Q-Sepharose anion exchange column (GE Healthcare). The recombinant TatD was then eluted with a high salt gradient containing a mixture of QA and QB buffer (20 mM MOPS, 1 mM β-mercaptoethanol, 1 M NaCl, pH 7.2) (gradient length: 100 ml). The purified TatD was concentrated to 3–9 mg/ml in 10 mM MOPS and 250 mM NaCl (pH 7.2). TatD point mutants were generated using the QuikChange site-directed mutagenesis kit (Stratagene, USA) and purified by the same procedures as those of wild-type TatD. The molecular weight of the wild-type TatD was determined by the Matrix-assisted laser desorption/ionization-Time-of-flight (MALDI-TOF) mass spectrometry (Bruker AutoFlex III smartbeam TOF/TOF200).

To determine the cellular location of TatD, the harvested *E. coli* cell pellet with overexpressed TatD was resuspended in Tris–sucrose hypertonic buffer (200 mM Tris–HCl, pH 8.0, 0.5 M sucrose and 0.5 mM Ethylenediaminetetraacetic acid (EDTA)) to a final concentration of 5–20 mg cells/ml. Lysozyme was added to the cell solution to a final concentration of 60 μg/ml to remove the cell wall. After incubation with lysozyme for 10 min, MgSO_4_ was added to a final concentration of 20 mM to stabilize the spheroplast which was then centrifuged at 8000*g* for 20 min. The supernatant was collected as the periplasmic fraction. The pellet contained spheroplast that was further resuspended and lysed by sonication and the supernatant was collected as the cytosol fraction.

### Nuclease activity assay

DNA and RNA oligonucleotides used for nuclease activity assays were labeled at the 5′ end with [γ-^32^P]ATP by T4 polynucleotide kinase and purified on a Microspin G-25 column (GE Healthcare) to remove the non-incorporated nucleotides. The 5′-end ^32^P-labeled 20-nucleotide single-stranded DNA or RNA (5′-CAAACTCTCTCTCTCTCAAC-3′ or 5′-CAAACUCUCUCUCUCUCAAC-3′) or the double-stranded DNA in a reaction buffer of 3 mM MOPS and 75 mM NaCl (pH 7.2) was incubated with the recombinant TatD in the presence or absence of metal ions in a final volume of 10 μl at 37°C for 1 or 2 h. The reactions were stopped by adding 20 mM protease K for 30 min to digest TatD. Reaction samples were then resolved on 20% denaturing polyacrylamide gels and visualized by autoradiography (Fujifilm, FLA-5000).

### Cell survival studies

Wild-type *E. coli* K-12 and TatD-knockout (ΔTatD) strain used in the survival studies were from the Keio collection (14). To measure the chronic sensitivity to 1 mM H_2_O_2_, 3 mM methyl methanesulfonate (MMS) and 120 nM mitomycin (MMC), serial dilutions of cells were spotted on plates containing indicated concentrations of the DNA damaging agents and incubated overnight at 37°C. To measure the sensitivity against UV-C, serial dilutions of cells were spotted on plates and exposed to UV-C (254 nm) in 20 J/m^2^ for 10 s by Hoefer UVC 500*-*Ultraviolet Crosslinker (Hoefer Inc). After UV-C irradiation, cells were incubated overnight at 37°C. To measure the acute sensitivity to hydrogen peroxide (H_2_O_2_), cells were exposed to 0, 10, 20, 40 and 80 mM H_2_O_2_ for 20 min. After removing H_2_O_2_, cells were diluted 100-fold into 10 ml LB medium and further grown on a rotary shaker (200 rpm) at 37°C for the measurement of *A*_600_ (OD) at 60 min intervals.

### Protein crystallization and structural determination

TatD was crystallized by the hanging-drop vapor diffusion method at 4°C and room temperature using a Hampton protein crystallization screen kit. The apo-form protein crystal was grown in the crystallization drop made by mixing of 1 μl protein sample (6–9 mg/ml TatD, 10 mM MOPS and 250 mM NaCl) and 1 μl reservoir solution (0.2 M ammonium citrate dibasic and 20% (w/v) polyethylene glycol 3350). TatD crystals appeared in 3 days with a size of ∼0.2 × 0.05 × 0.01 mm^3^.

For the co-crystallization of TatD with DNA, TatD was mixed with the single-stranded DNA 5′-GCTTAGCT-3′ at a molar ratio of 1:1.2. TatD–DNA co-crystals were grown by mixing 1 μl TatD–DNA mixture (7–8 mg/ml TatD, 10 mM MOPS and 250 mM NaCl) and 1 μl reservoir solution (4% Tacsmate at pH4 and 12% (w/v) polyethylene glycol 3350). TatD–DNA complex crystals appeared in 3–7 days with a size of ∼0.08 × 0.08 × 0.01 mm^3^.

Single-wavelength X-ray diffraction data of TatD were collected using synchrotron radiations at the BL13B1 beamline at NSRRC, Taiwan. Data integration and scaling were performed using HKL2000. The reflection phases were determined by molecular replacement using the crystal structure of the zinc-bound *E. coli* TatD (PDB ID: 1XWY) as the search model by the PHENIX program. The structure model was further built and refined by Wincoot and PHENIX, respectively. Structural coordinates and diffraction structure factors have been deposited in the RCSB Protein Data Bank with the PDB ID codes of 4P5U for the apo-form TatD and 4PE8 for the DNA-bound TatD. The diffraction and refinement statistics are listed in Table 1.

**Table 1.** X-ray data collection and refinement statistics for the crystal structures of TatD and TatD–DNA complex

**Data collection statistics**		
Crystal	TatD	TatD–DNA
Wavelength (Å)	1	1
Space group	*P*2_1_2_1_2_1_	*P*2_1_2_1_2_1_
Cell constants (*a*, *b*, *c*) (Å)	*a* = 43.2, *b* = 52.7, *c* = 98.4	*a* = 43.5, *b* = 52.9, *c* = 101.1
Resolution (Å)	30–2.0 (2.03–2.0)	30–2.9 (3–2.9)
Observed/unique reflections	90 131/15 670	30 630/5539
Data redundancy	5.8 (5.9)	5.5 (5.5)
Completeness (%)	98.8 (100.0)	99.4 (99.6)
*I*/*σ*(*I*)	23.2 (4.9)	17.9 (4.9)
Rsym (%)	7.8 (35.3)	11.7 (42.8)

**Refinement statistics**		
Resolution	30–2.0	30–2.9
*R*-work/*R*-free	20.3/24.2	19.5/24.0
Number of atoms (protein/water/DNA)	2055/219/0	2045/39/58
Average *B*-factor (protein/water/DNA) (Å^2^)	21.5/27.6/0	40.8/36.7/69.2
RMSD in bond length (Å)/bond angle (°)	0.004/0.98	0.003/0.72

Values in parentheses refer to the highest resolution shell.

## RESULTS

### *E. coli* TatD is a Mg^2+^-dependent 3′–5′ exonuclease specific for single-stranded DNA and RNA

**Figure 2.**
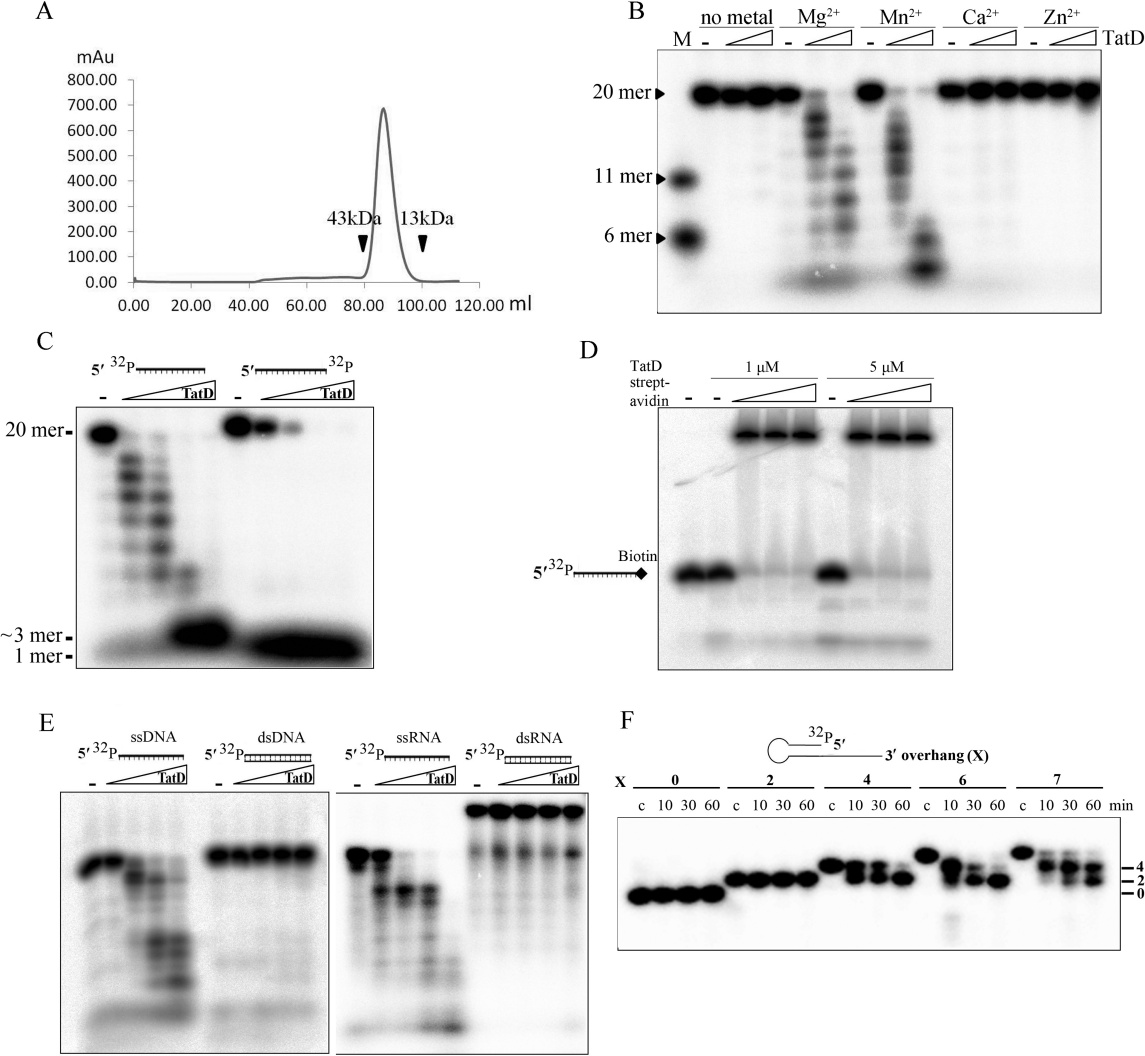
*E. coli* TatD is a Mg^2+^-dependent 3′–5′ exonuclease specific for single-stranded DNA and RNA. (**A**) The gel filtration (Superdex 200 16/60 PG) profile of the full-length TatD shows that the enzyme was eluted as a monomeric protein. (**B**) TatD (0–0.5 μM) was incubated with 5′ end ^32^P-labeled 20-nucleotide ssDNA (40 nM) for 1 h at 37°C. TatD cleaved DNA most efficiently in the presence of Mg^2+^ or Mn^2+^ whereas TatD had no nuclease activity with the presence of Ca^2+^ or Zn^2+^. The final cleaved products were small DNA of less than six nucleotides. (**C**) TatD (0–0.5 μM) produced a ladder pattern of digested products in digesting 5′ end ^32^P-labeled ssDNA (40 nM) whereas it produced a single band of 3′-^32^P-NMP in digesting 3′-^32^P-labeled DNA. (**D**) TatD could not digest the 3′-biotin-bound DNA in the presence of streptavidin, which blocked access of TatD from the 3′ end. (**E**) TatD preferred to digest single-stranded DNA/RNA over double-stranded DNA/RNA. (**F**) TatD (1 μM) digested the 3′ overhang of a duplex DNA (40 nM) to generate a final product of a duplex with a 2-nucleotide 3′ overhang. The number of nucleotides in the 3′ overhang are marked at the right of the gel.

To characterize the biochemical properties and examine the structural basis of TatD in DNA degradation, the full-length *E. coli* TatD with a C-terminal His-tag was expressed and purified by chromatographic methods using a Ni-NTA affinity column followed by a Q-Sepharose anion exchange column. The recombinant TatD had a high homogeneity as revealed by the sodium dodecyl sulfate polyacrylamide gel electrophoresis (SDS-PAGE) (Supplementary Figure S1B). The gel filtration profile showed that TatD formed a stable monomer with a molecular weight of ∼30 kDa at pH 7.2 (Figure 2A). Mass spectroscopy indicated that the purified TatD was a full-length protein with a molecular weight of 30 016 Da (compared with the calculated molecular weight of 30 039 Da).

To characterize the nuclease activity, the recombinant TatD was incubated with the 5′-end ^32^P-labeled 20-nucleotide single-stranded DNA in the presence of different divalent metal ions. The DNA digestion assay showed that TatD cleaved DNA in the presence of Mg^2+^ (2 mM) and Mn^2+^ (2 mM) but was without detectable nuclease activity in the presence of Ca^2+^ (1 mM) or Zn^2+^ (100 μM) or no ion (Figure 2B). This result confirmed the earlier finding showing that yeast TatD is a Mg^2+^-dependent DNase (10). To characterize the exo- or endonuclease activity of TatD, we prepared a 5′-^32^P-labeled and a 3′-^32^P-labeled single-stranded DNA for TatD digestion. TatD produced a ladder pattern of cleaved products following digestion of 5′-^32^P-labeled DNA but only a single band of 3′-^32^P-NMP without any middle band in digestion of 3′-^32^P-labeled DNA, suggesting that TatD is a 3′–5′ exonuclease (Figure 2C). Moreover, we estimated that the final cleaved products by TatD had a size of about three nucleotides as they were more than one nucleotide as shown in Figure 2C and less than six nucleotides as shown in Figure 5B. To further confirm the 3′–5′ exonuclease activity, we found that TatD could not digest the 3′-biotin-bound DNA in the presence of streptavidin, which blocked the access of TatD from the 3′ end (Figure 2D). These results thus show that TatD is a 3′–5′ exonuclease without any endonuclease activity in digesting single-stranded DNA.

**Figure 3.**
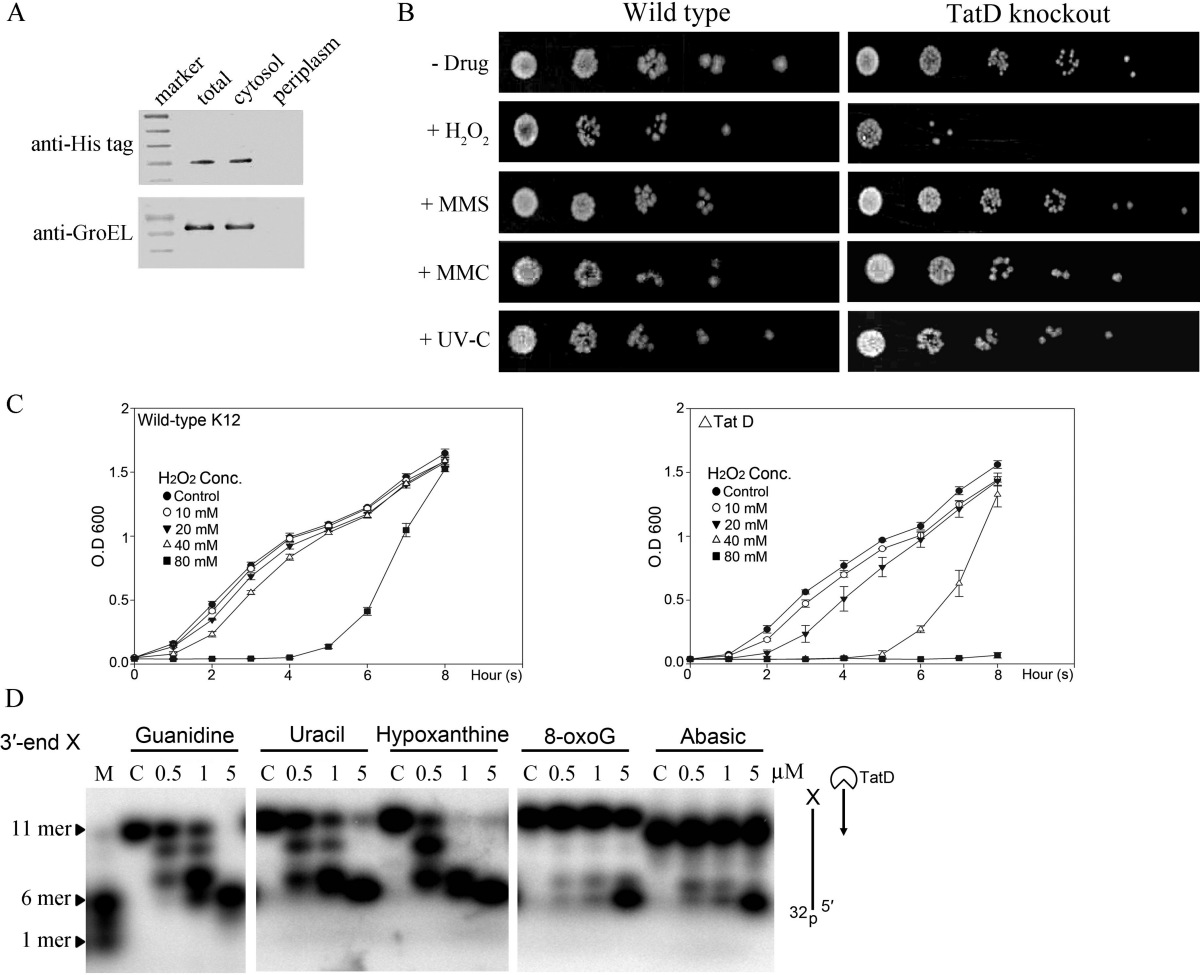
TatD is a DNA repair exonuclease in *E. coli*. (**A**) The *E. coli* (BL21-CodonPlus-RIPL) cell extracts isolated from different compartments reveal that the overexpressed His-tagged TatD is located in cytoplasm. (**B**) Wild-type *E. coli* K-12 strains were resistant to chronic doses of various DNA damaging agents, including hydrogen peroxide (1 mM H_2_O_2_), methyl methanesulfonate (3 mM MMS), mitomycin C (120 nM MMC) and UV-C light (20 J/m^2^ for 10 s). The TatD-knockout strain was sensitive to H_2_O_2_ but not MMS, MMC and UV-C radiation. (**C**) Growth curves of wild-type and TatD knockout cells (ΔTatD) after acute exposure to H_2_O_2_ (10–80 mM) for 20 min. (**D**) The single-stranded DNA containing a deaminated, oxidized base or an abasic site at the 3′-terminal end (5′-GAGTCCTATA**X**-3′) were incubated with TatD in the DNA digestion experiment. TatD digested efficiently the single-stranded DNA with a 3′-terminal deaminated base (uracil, hypoxanthine), however, the single-stranded DNA with a 3′-terminal oxidized base, 8-oxoguanine (8-oxoG) or an abasic site were more resistant to TatD.

**Figure 4.**
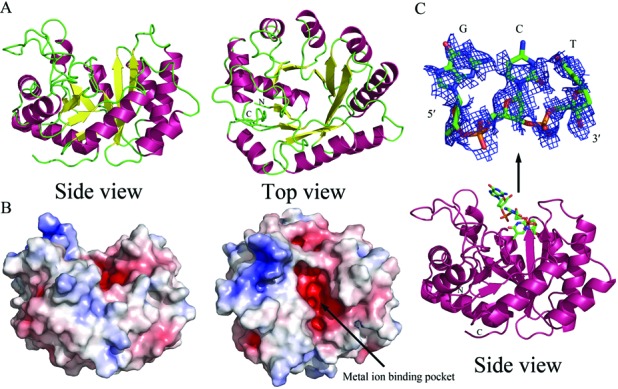
Crystal structures of the apo-form and DNA-bound TatD. (**A**) The ribbon model (side and top view) of the crystal structure of the apo-form TatD with a TIM-barrel fold. (**B**) The electrostatic surface potential of TatD reveals an acidic metal-ion-binding pocket on the top of the TIM-barrel with a basic DNA-binding surface on the left side. (**C**) The crystal structure of TatD bound with a tri-nucleotide (5′-G-p-C-p-T-3′) DNA reveals that DNA is bound on the top loop region of the TIM-barrel. The magnification of the omit map (2*F*_o_ − *F*_c_, 0.3*σ*) of the tri-nucleotide is shown on the panel above. The Fourier and difference Fourier maps are displayed in the supplementary Figure S1A. The two highest peaks in the difference map were assigned as the two phosphate groups in the tri-nucleotide 5′-G-p-C-p-T-3′.

**Figure 5.**
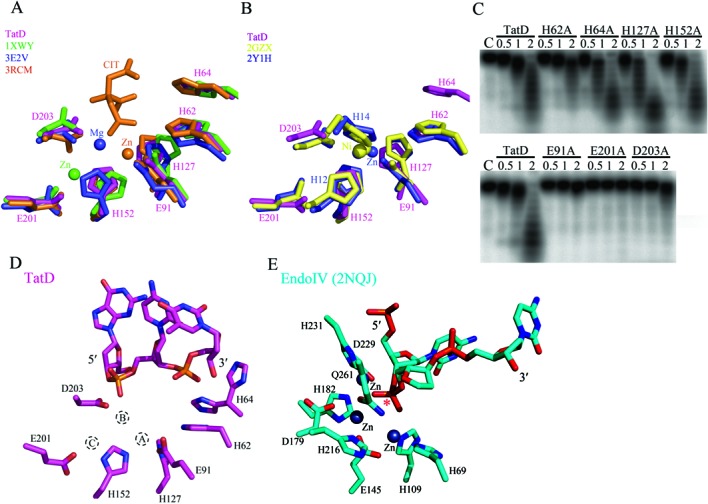
The conserved metal ion-binding and catalytic residues in the active site of TatD. (**A**) The superimposition of the active site of the metal-free TatD (this study, 4P5U) with one-metal ion TatD from *E. coli* (1XWY, Zn^2+^), *Pseudomonas putida* (3RCM, Zn^2+^) and *S.cerevisiae* (3E2V, Mg^2+^) reveals five conserved metal ion-binding residues, E91, H127, H152, E201 and D203. CIT represents the citric acid molecule bound in the crystal structure of *P. putida* TatD. (**B**) The superimposition of the active site of the metal-free TatD (this study) with two-metal ion TatD from *S. aureus* (2GZX, 2 Ni^2+^) and *H. sapiens* (2YIH, 2 Zn^2+^) reveals that the two metal-ion binding histidines, H12 and H14, are absent in *E. coli* TatD. (**C**) The exonuclease activity assays for the wild-type and single-point mutated TatD reveal that H64A, H127A and H152A retained their nuclease activity, H62A had a significant reduced exonuclease activity, and E91A, E201A and D203A lost their exonuclease activity in digestion of a 20-nucleotide single-stranded DNA. (**D**) The putative active site in TatD–DNA complex reveals a number of conserved histidine and acidic residues. The metal A shown here is the Zn^2+^ site in *P. putida* TatD whereas the metal B is the Mg^2+^ site of yeast TatD, and the metal C is the Zn^2+^ site in *E. coli* TatD. The side chain of H64 residue has two alternative conformations. (**E**) The active site of Endo IV shares little resemblance to that of TatD with three bound zinc ions (blue sphere). The star marks the scissile phosphate in the double-stranded DNA bound on Endo IV.

To determine the substrate preference of TatD, we incubated TatD with 5′-^32^P-labeled single-stranded and double-stranded DNA, as well as single-stranded and double-stranded RNA. TatD almost completely digested the single-stranded DNA and RNA whereas it was unable to digest the double-stranded DNA and RNA (Figure 2E). This result suggests that TatD prefers to cleave single-stranded over double-stranded nucleic acids. We also found that TatD digested the 3′ overhang of more than three nucleotides of a duplex DNA to generate a final product of a duplex with a 2-nucleotide 3′ overhang (Figure 2F). The short overhang generated by TatD was correlated to the short final products of ∼3 nucleotides generated by TatD in the digestion of single-stranded DNA (see Figure 3C). In summary, these results suggest that TatD is a 3′–5′ exonuclease that can trim not only the 3′ end of a single-stranded DNA but also the 3′ overhang of a duplex DNA.

### TatD is a DNA repair exonuclease

To characterize the cellular function of TatD, we first isolated different fractions of cell extracts and found that TatD was located in the cytoplasm in *E. coli* (Figure 3A). This result is consistent with the earlier finding showing that TatD is not a periplasmic protein as are other Tat proteins in *E. coli* (3). Based on the biochemical properties of TatD, a 3′–5′ exonuclease specific for single-stranded DNA located in cytoplasm, it is likely that TatD may process DNA in DNA repair pathways. To test this hypothesis, we treated wild-type *E. coli* K-12 strain and TatD-knockout cells with different DNA damaging agents, including H_2_O_2_, MMS, MMC and UV light. The wild-type K-12 strain resisted all DNA damaging agents when present at a chronic dose whereas the TatD-knockout strain was sensitive to the chronic dose of H_2_O_2_ (Figure 3B). The sensitivity of the TatD deficient strain to H_2_O_2_ was further confirmed by treatment of cells with an acute dose of H_2_O_2_ at various concentrations from 10 to 80 mM for 20 min. After H_2_O_2_ treatment (40 and 80 mM), the TatD-knockout strain (ΔTatD) grew slower or could not grow as compared to those of wild-type K12 strain (Figure 3C). These results support that TatD may be involved in the H_2_O_2_-induced DNA damage repair.

Hydrogen peroxide may induce various DNA lesions, including oxidation and deamination of bases and sugar modifications (15,16). To further characterize the role of TatD in DNA repair pathways, a single-stranded DNA containing a deaminated or oxidized base or an abasic site at the 3′-terminal end of the single-stranded DNA 5′-GAGTCCTATAX-3′ were incubated with TatD. We found that TatD digested more efficiently the DNA with a deaminated base (uracil and hypoxanthine) but less efficiently the DNA with a 3′-terminal oxidized base (8-oxoguanine, 8-oxoG) and an abasic site that were more resistance to TatD digestion (Figure 3D). This result suggests that TatD can function as an exonuclease in the excision step for deaminated bases in the H_2_O_2_-induced DNA repair.

### Crystal structure of TatD and TatD–DNA complex

To provide structural insights into TatD, we crystallized the apo-form TatD by the hanging-drop vapor diffusion method and determined the crystal structure at a resolution of 2.0 Å by molecular replacement using the zinc-bound *E. coli* TatD (PDB ID: 1XWY) as the search model (Figure 4A). The X-ray diffraction and structural statistics are listed in Table 1. TatD has a TIM-barrel fold, forming a barrel made of eight β-strands surrounding by nine α-helices. The electrostatic surface potential of TatD revealed an acidic pocket on the top loop region of the β barrel with a patch of basic surface next to the pocket (Figure 4B). This acidic pocket is likely the metal-ion binding site and the basic surface on the side with several basic residues, including R98, R129 and R219, is likely involved in DNA binding.

To understand how TatD binds and cleaves DNA, we further co-crystallized TatD with a single-stranded DNA (5′-GCTTAGCT-3′). TatD–DNA complex was crystallized by the hanging-drop vapor diffusion method and the co-crystal diffracted X-ray to a resolution of 2.9 Å. The structure of the complex was determined by molecular replacement and the structural statistics are listed in Table 1. The simulated annealing omit Fourier map (2*F*_o_ − *F*_c_) of the bound DNA revealed weak electron density for the three nucleotides (5′-G-p-C-p-T-3′) (Figure 4C, top panel). We modeled the DNA with the two phosphates positioned at the two peak heights in the difference Fourier map (Supplementary Figure S1A). The 8-nucleotide single-stranded DNA was likely digested by TatD and resulted a 3-nucleotide DNA. Alternatively, it is possible that the 5′ end of the DNA was disordered and only the three nucleotides at the 3′ end were observed. The DNA was refined with an occupancy of 0.7 and an averaged *B*-factor of 69.2 Å^2^ and was bound at the loop region on the top of the TIM-barrel in a way similar to that of Endo IV (13).

Superimposition of the apo-form and DNA-bound TatD gave an average RMSD of 0.394 Å for 259 Cα atoms, suggesting that DNA binding did not induce any major conformational change. However, a close look at the protein–DNA interface showed that the loop with R98 and F100 moved toward DNA in the TatD–DNA complex structure, and as a result, the two residues, R98 and F100 were disordered and refined with an occupancy of 0.79 and 0.85, respectively (Supplementary Figure S2A). Moreover, the residue H64 near the scissile phosphate had two alternative conformations and was refined with an occupancy of 0.53 (apo-form conformation) and 0.47 (flip-out conformation), respectively. The side-chain of H64 and R98 rotate their conformations and directly interact with the DNA backbone (Supplementary Figure S2C). However, no metal ion was identified in the TatD–DNA complex structure and DNA is bound loosely between two TatD molecules in the crystal (see Supplementary Figure S2B), indicating that the 5′-G-p-C-p-T-3′ was bound in an inactive conformation. It is likely that the crystal structure of TatD–DNA complex reported here represents the conformation of TatD bound with a cleaved DNA product.

A structural homology search by DALI revealed that our apo-form TatD structure bears a high structural resemblance to the TatD structures in the protein data bank, including Zn-bound *E. coli* TatD (PDB entry: 1XWY, an average RMSD of 0.72 Å for 256 Cα atoms), human TatD1 (2XIO, 1.30 Å for 241 Cα atoms), human TatD3 (2YIH, 1.51 Å for 237 Cα atoms) and yeast TatD (3E2V, 1.54 Å for 245 Cα atoms). TatD also shares a high structural resemblance to the metal-dependent hydrolases with a TIM-barrel fold (12), such as phosphotriesterase homology protein (1BF6, 2.55 Å for 220 Cα atoms), urease (1EJV, 2.99 Å for 200 Cα atoms) and adenine deaminase (2ICS, 2.59 Å for 202 Cα atoms). The TIM-barrel endonuclease, Endo IV, shares a low sequence identity (7.3%) with TatD, however, the structure of Endo IV (2NQH) also closely resemble that of TatD with an RMSD of 3.91 Å for 164 Cα atoms. These results suggest that TatD can be classified as one of the TIM-barrel enzymes in the subfamily of metal-dependent hydrolases.

### Active site of TatD

To reveal the catalytic mechanism, we further superimposed and compared the active site residues of TatD with its homologues (Figure 5A). Interestingly, the superimposition revealed two sub-groups of TatD, Group I with a single metal ion and Group II with two metal ions coordinated in the active site. The Group I TatD from *E. coli* (1XWY, Zn^2+^), *Pseudomonas putida* (3RCM, Zn^2+^) and *S. cerevisiae* (3E2V, Mg^2+^) have five conserved metal ion-binding residues, E91, H127, H152, E201 and D203 (see Figure 5A). Since Mg^2+^ activates the exonuclease activity of TatD, the Mg^2+^ ion in the yeast TatD (3E2V) may represent the single metal ion-binding state of TatD without a bound substrate. However, upon substrate binding, it is likely that two or three metal ions are bound in the active site, since the superimposition revealed three different putative metal ion-binding sites in TatD (see Figure 5A).

Superimposition of our TatD with the Group II subfamily of TatD with two transition metal ions, *S. aureus* TatD (2GZX, 2Ni^2+^) and human TatD3 (2YIH, 2Zn^2+^), further revealed two conserved metal-binding histidine residues that are absent in the Group I TatD (Figure 5B). These two histidines (H12 and H14 in 2YIH) together with other histidine residues in the active site form histidine clusters for binding of two transition metal ions, Zn^2+^ or Ni^2+^. Since the *E. coli* TatD does not bear these two histidine residues, very likely it belongs to the Group I TatD that are activated by Mg^2+^ but not by Zn^2+^. Therefore, based on the structure comparison of the active site of TatD, *E. coli* TatD resembles more closely to the group I TatD and likely binds two or three Mg^2+^ ions in the active site for DNA hydrolysis.

To further identify the catalytic residues in TatD for DNA hydrolysis, we mutated the conserved residues located in the active site and constructed seven single-point mutants, including H62A, H64A, E91A, H127A, H152A, E201A and D203A. We found that most of the histidine mutants, including H64A, H127A and H152A, retained their nuclease activity, except that H62A had a significantly reduced exonuclease activity, in comparison to the wild-type TatD (Figure 5C, top panel). In contrast, mutation of the acidic residues, including E91A, E201A and D203A, produced inactive mutants with little residual exonuclease activity (Figure 5C, bottom panel). This result suggests that the three acidic residues, E91, E201 and D203, are involved in binding of two to three magnesium ions in the active site (see Figure 5D). In summary, we suggest that the conserved acidic residues E91, E201 and D203 are responsible for binding of metal ions, and H62 for playing a critical catalytic role in the active site of *E. coli* TatD in DNA hydrolysis.

## DISCUSSION

### How a TIM-barrel protein functions as an exonuclease

The TIM-barrel fold, first seen in triosephosphate isomerase, is the most common enzyme fold of known protein structures (17). TIM-barrel folds have been identified in many different enzyme families with diverse catalytic functions (12). The structural-homology search by DALI suggests that TatD shares significant structural similarity to the members of metal-dependent hydrolases that hydrolyze different substrates, such as phosphotriesterase homology protein hydrolyzes organophosphate triesters, urease hydrolyzes urea and adenine deaminase hydrolyzes adenine. TatD represents the first example of a TIM-barrel exonuclease that hydrolyzes the phosphodiester bond at the 3′ end of a nucleic acid chain.

How does a TIM-barrel exonuclease hydrolyze DNA? A superposition of TatD–DNA and Endo IV–DNA reveals that the TatD-bound nucleotides fit at the similar location as those bound on the Endo IV (Supplementary Figure S3). The 3′-end phosphate of the TatD-bound DNA is located near the scissile phosphate of the Endo IV-bound DNA with a distance of 5.2 Å. However, Endo IV binds a long duplex DNA that extends from the left to the right on the top of the TIM-barrel. On the other hand, the single-stranded DNA should be extended only to one side on the top of the TIM-barrel TatD. This is indeed consistent with the surface potential of TatD which is basic only on the left top region (see Figure 4B). Thus, the single-stranded DNA is bound on one side on the top of the TIM-barrel and therefore the 3′ end of DNA inserts into the active site of TatD for hydrolysis (see the DNA-binding model in the Supplementary Figure S2D).

Although the overall TIM-barrel fold of TatD is similar to that of Endo IV, the active sites of the two enzymes share little resemblance (Figure 5D and E). Endo IV has three zinc ions in the active site with the Glu261 functioning as the general base to deprotonate a zinc-bridging water molecule for the nucleophilic attack on the scissile phosphate (18). The three zinc ions in Endo IV do not match at the same locations as the metal ions bound in the active site of TatD homologues (see Supplementary Figure S3C). TatD might bind two or three magnesium ions and hydrolyze DNA via a two-metal ion or three-metal ion catalytic pathway (19). Alternatively, the metal ions may move as seen in RNase H (20) or be transiently bound during hydrolysis as observed in the DNA polymerases (21,22). Without the accurate number and location of the metal ions and the DNA substrate bound in the active conformation, it is challenging to predict how the DNA is cleaved by TatD. Moreover, the recombinant TatD had a low enzyme activity in our assays (Figures 2 and 3), suggesting that other unknown co-factors are probably involved in DNA hydrolysis. Further studies are required to determine the hydrolysis mechanism catalyzed by TatD.

### TatD in DNA repair

*Escherichia coli* has a number of 3′–5′ exonucleases that play a role in DNA repair, including ExoI (23,24), ExoIII (25), ExoVII (23), ExoIX (26), ExoX (24), PNPase (15,27) and RNase T (28). These exonucleases have different substrate specificities and are involved in different DNA repair pathways. ExoI, ExoVII and ExoX are specific for single-stranded DNA in mismatch and DNA recombination repair pathways (23,29,30). ExoIII bearing not only 3′–5′ exonuclease, but also 3′-phosphomonoesterase and AP endonuclease activity, is suggested to play a role in base excision repair (23). On the other hand, RNase T trims the 3′ end of structured DNA, including bulge, bubble and Y-structured DNA, in Endonuclease V-dependent, UV-induced and other DNA repair pathways (28).

Here we show that TatD is specific for trimming single-stranded DNA and the 3′ overhang of a duplex DNA. This result is consistent with the earlier finding showing that TatD limits RecA loading onto single-stranded DNA substrates and TatD functions in a common pathway with ExoVII, ExoIX and ExoX in negatively regulating the RecA-dependent homologous recombination by degrading RecA substrates (31). We show here that TatD is involved in H_2_O_2_-induced DNA repair that include double-strand break repair. Taken together these lines of evidence, we suggest that TatD digests single-stranded DNA and is involved in the regulation of double-strand break repair. Hydrogen peroxide may induce various DNA lesions, not only double-strand breaks, but also oxidation and deamination of bases and sugar modifications (15,16). We show here that the TatD-knockout *E. coli* is sensitive to H_2_O_2_, and TatD can remove the deaminated nucleotide from a DNA chain. Therefore, in addition to double-strand break repair, our biochemical and *in vivo* data suggest that TatD may also process the 3′ ends of DNA in the nucleotide excision repair.

In conclusion, based on *in vivo* and biochemical evidence, we identified TatD as a member of a new group of DNA repair exonucleases. TatD has a TIM-barrel fold and it trims DNA from 3′-to-5′ ends in the hydrogen peroxide-induced DNA repair, including double-strand break repair and nucleotide excision repair in *E. coli*. Our results suggest that TatD not only digests chromosomal DNA during apoptosis but also processes damaged DNA during DNA repair. This finding opens a new direction for the study of the TatD homologues in various species from bacteria to human.

## ACCESSION NUMBERS

PDB entry codes: 4P5U, 4PE8.

## SUPPLEMENTARY DATA

Supplementary Data are available at NAR Online.

SUPPLEMENTARY DATA
