# Supplementary material for: Structure and function of TatD exonuclease in DNA repair
Source: Nucleic Acids Res. 2014 Aug 11;42(16):10776–85. doi: 10.1093/nar/gku732 (PMC4176340; doi:10.1093/nar/gku732)
Supplement: SUPPLEMENTARY DATA [file supp_42_16_10776__index.html]

Structure and function of TatD exonuclease in DNA repair — Structure and function of TatD exonuclease in DNA repair — SUPPLEMENTARY DATA 

# Structure and function of TatD exonuclease in DNA repair

## SUPPLEMENTARY DATA

**Files in this Data Supplement:**

- SUPPLEMENTARY DATA
