# Supplementary material for: Structure and function of TatD exonuclease in DNA repair
Source: Nucleic Acids Res. 2014 Aug 11;42(16):10776–85. doi: 10.1093/nar/gku732 (PMC4176340; doi:10.1093/nar/gku732)
Supplement: SUPPLEMENTARY DATA [file supp_gku732_nar-01089-z-2014-File007.pdf]

Supplementary Information
for
Structure and function of TatD exonuclease in DNA repair

Yi-Jen Chen[1&], Chia-Lung Li[1&], Yu-Yuan Hsiao[2], Yulander Duh[1] and Hanna S. Yuan[1,3*]

[1]Institute of Molecular Biology, Academia Sinica, Taipei, Taiwan 11529, ROC.
[2]Department of Biological Science and Technology, National Chiao Tung University, Hsinchu, Taiwan 30068, ROC.
[3]Graduate Institute of Biochemistry and Molecular Biology, National Taiwan University, Taiwan 10048, ROC.
[&]These two authors contributed equally to the work.

This file include:
Supplementary Figure S1, S2 and S3.

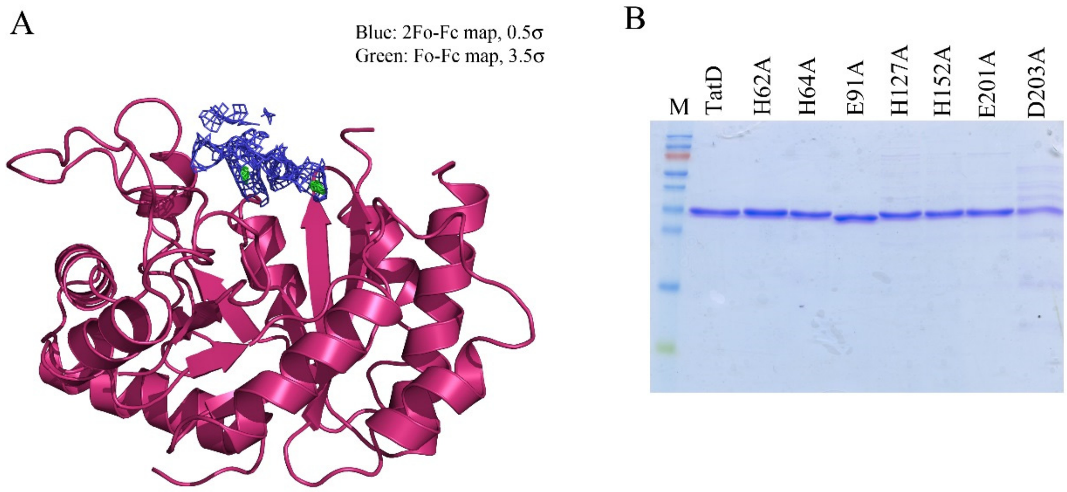

Supplementary Figure S1. Structure of TatD-DNA complex and the constructed TatD mutants. (A) The Fourier map of the tri-nucleotide 5'-G-p-C-p-T-3' in the crystal structure of TatD-DNA complex. The Fourier maps calculated without the inclusion of any DNA in the model revealed weak electron density: 0.5 σ cut off in the (2Fo-Fc) map (blue) and 3.5 σ cut off in the (Fo-Fc) map (green). The two highest peaks in the maps were assigned as the two phosphate groups in the tri-nucleotide 5'-G-p-C-p-T-3'. (B) Seven TatD single-point mutants were constructed: H62A, H64A, E91A, H127A, H152A, E201A and D203A. Molecular weight of TatD mutants were confirmed by Mass spectrometry (data not shown). The SDS-PAGE revealed the high homogeneity of the recombinant wild-type and mutated TatD.

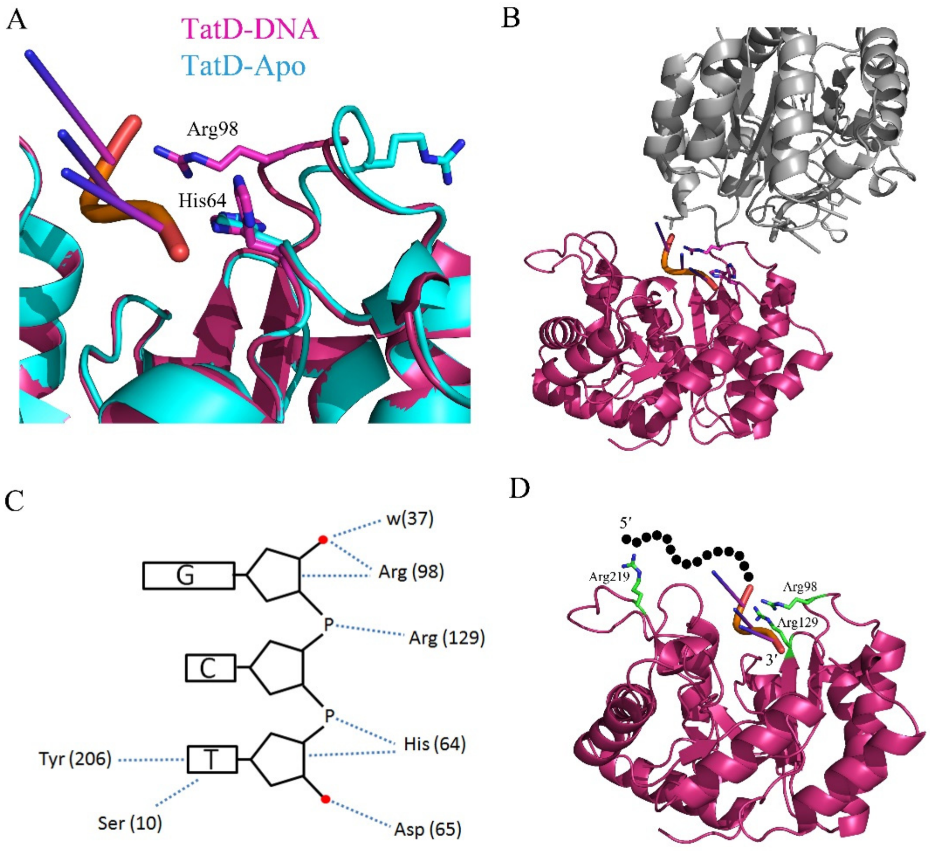

Supplementary Figure S2. The interactions between TatD and DNA. (A) The superimposition of TatD with TatD-DNA structures reveals that the loop with Arg98 moves toward DNA and the side chain of His64 flips out upon DNA binding. (B) The tri-nucleotide in the TatD-DNA complex was bound between two TatD molecules, indicating that the tri-nucleotide was likely a cleaved product that was trapped in the crystals. (C) The schematic diagram shows the hydrogen bonding and van der Waals interaction networks between TatD and DNA. (D) The 5′ end of the single-stranded DNA are likely extended to further interact with the basic region near Arg219 on the left top side of TatD.

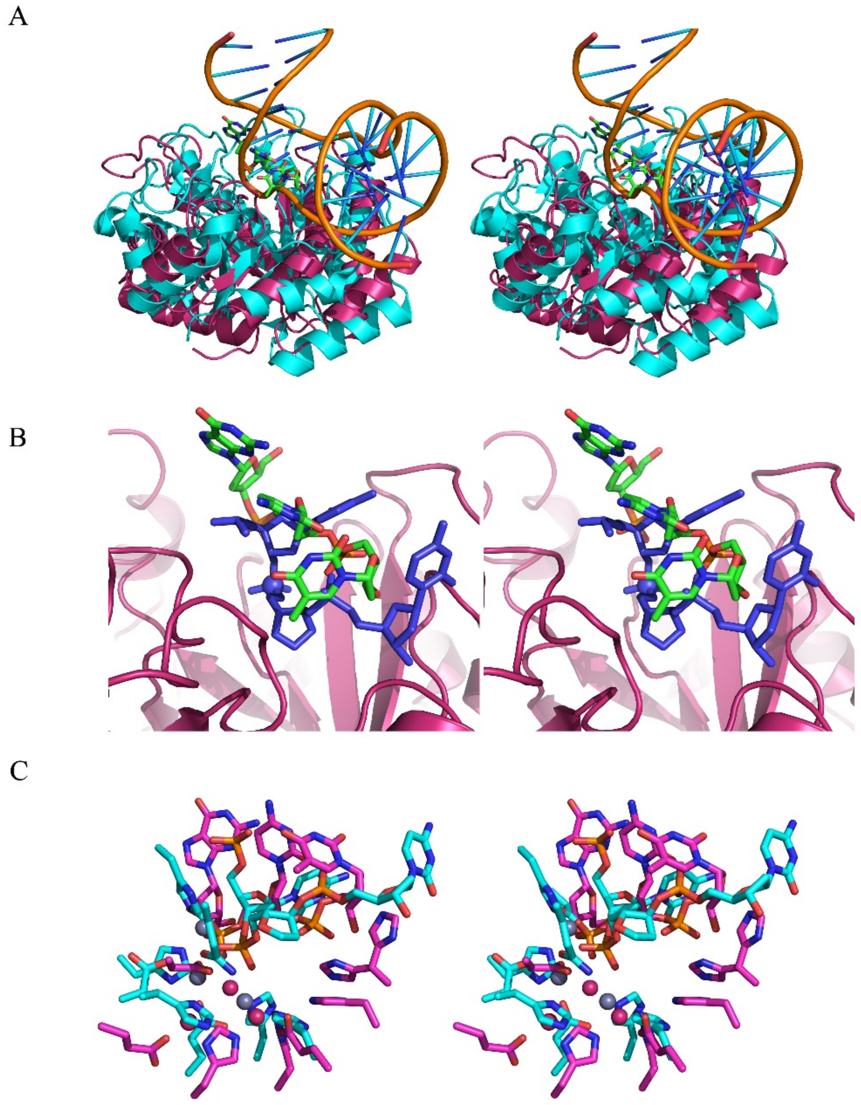

Supplementary Figure S3. Comparison of the crystal structures of TatD-DNA and Endo IV-DNA complex. (A) The stereo view of the superimposition between TatD (magenta) and EndoIV (cyan) revealed a similar TIM barrel fold with an average RMSD of 3.91 Å for 164 Cα atoms. The TatD-associated tri-nucleotide (in green) is bound on the top of the TIM barrel at the similar location to the Endo IV-bound duplex DNA. (B) The stereo view of the superimposition of Endo IV-bound DNA (2NQJ) to the crystal structure of TatD-DNA complex. Only 3 nucleotides (in blue) in the Endo IV-associated DNA are shown with the scissile phosphate marked in a ball. (C) The stereo view of the superimposed active site of TatD (magenta, PDB entry: 4PE8) with that of EndoIV (cyan, PDB entry: 2NQJ). The three zinc ions in EndoIV are displayed in gray spheres. For a comparison, the metal ions in magenta are adopted from yeast TatD (3E2V, Mg^{2+}), *P. putida* TatD (3RCM, Zn^{2+}), and *E. coli* TatD (1XWY, Zn^{2+}).
